# Sequence diagram refactoring using single and hybridized algorithms

**DOI:** 10.1371/journal.pone.0202629

**Published:** 2018-08-22

**Authors:** Abdulrahman Ahmed Bobakr Baqais, Mohammad Alshayeb

**Affiliations:** Information and Computer Science Department, King Fahd University of Petroleum & Minerals, Dhahran, Saudi Arabia; UCLA, UNITED STATES

## Abstract

Data mining and search-based algorithms have been applied to various problems due to their power and performance. There have been several studies on the use of these algorithms for refactoring. In this paper, we show how search based algorithms can be used for sequence diagram refactoring. We also show how a hybridized algorithm of Kmeans and Simulated Annealing (SA) algorithms can aid each other in solving sequence diagram refactoring. Results show that search based algorithms can be used successfully in refactoring sequence diagram on small and large case studies. In addition, the hybridized algorithm obtains good results using selected quality metrics. Detailed insights on the experiments on sequence diagram refactoring reveal that the limitations of SA can be addressed by hybridizing the Kmeans algorithm to the SA algorithm.

## Introduction

Changes and modifications to software are inevitable. Software organizations strive to improve their code or adjust it for a new platform, technology or structure. Refactoring is the process that shows how software can be improved without altering its behavior [1]. To perform refactoring, a mechanism should be devised to detect an anomaly or ill-structured piece in the software in order to improve it. This is usually accomplished via different approaches but the principle one in the literature is software metrics [2, 3]. Similar to code refactoring, software model refactoring, especially UML, has increasingly captured the interest of researchers to identify ill-structured components [4–7] and refactor them [8, 9].

**Fig 1:**
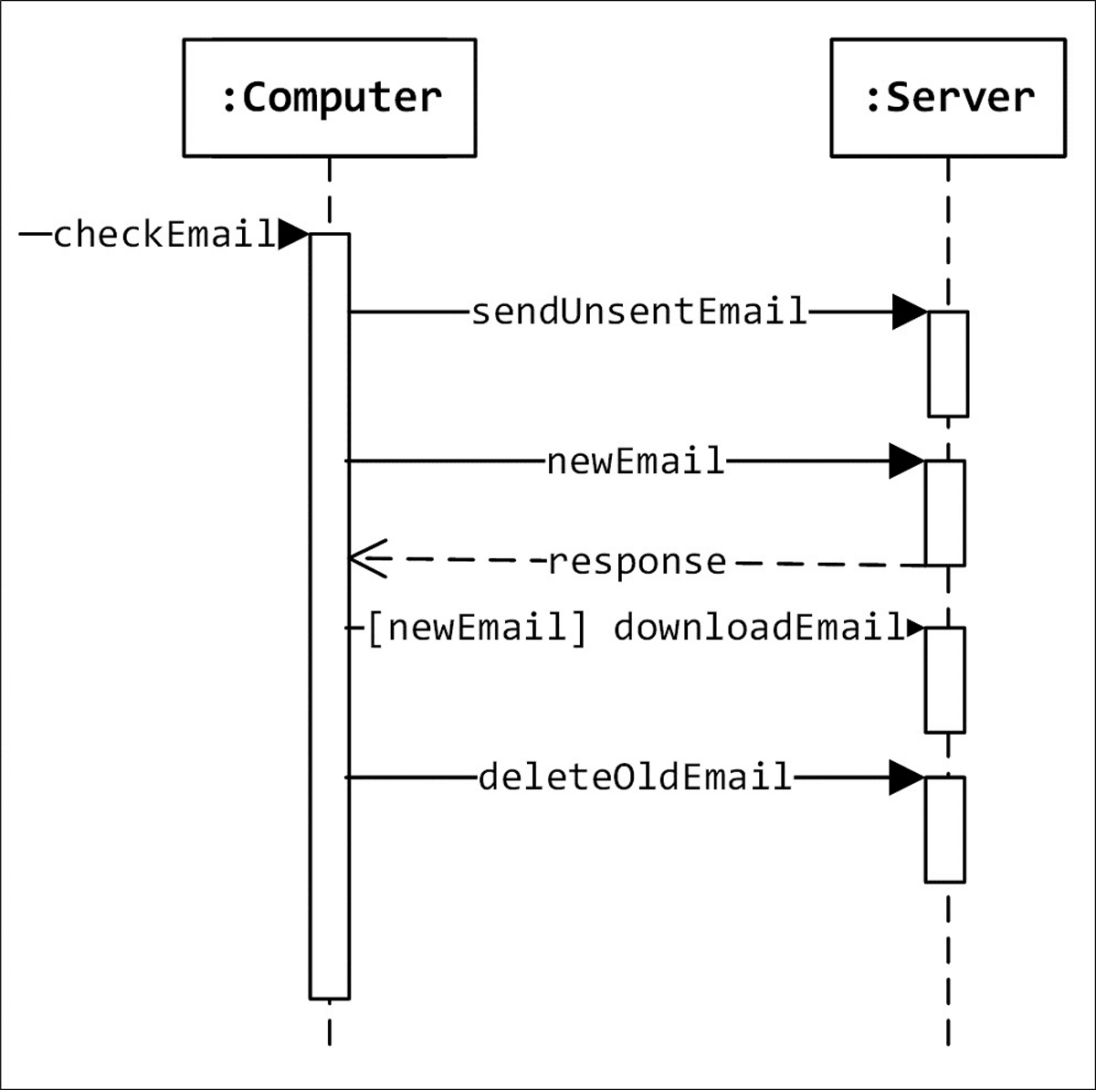
An example of a sequence diagram.

A sequence diagram is defined by the UML Reference Manual as “a diagram that shows object interactions arranged in time sequence. In particular, it shows the objects participating in an interaction and the sequence of messages exchanged” [10]. A sequence diagram is a dynamic UML diagram that shows the interaction between the components of the system. A sequence diagram shows how different objects of the system interact over time via messages. It represents objects as vertical lines and messages as arrows with labels. A sequence diagram is not intended to depict complex systems due to their extensive detail. Nevertheless, it is useful for developers because it increases the level of understanding of how different objects are implemented in the system. A sequence diagram can be considered as a protocol definition of certain tasks [11]. Usually, a sequence diagram is not large and it should correspond to one scenario only. Fig 1 shows an example of a sequence diagram.

Since the introduction of the search-based algorithm in software engineering [12], many papers have investigated the use of search-based algorithms in software refactoring [13–16]. Deterministic data mining techniques have also been considered in software refactoring. [17]. To overcome the limitations exist in deterministic data mining algorithms when applied to software refactoring, researchers proposed hybridized techniques [18, 19].

This paper tends to provide an automatic refactoring of sequence diagram using SA and also introduces a hybridized algorithm composed of search-based algorithm combined with a data-mining algorithm. Sequence diagrams depict the communication between different program components. Improper design of coupling and cohesion in sequence diagrams will propagate to the source code. This results in having methods with high number of parameters and methods that communicate with different classes. Hence, refactoring the sequence diagram helps designers in producing better code.

The aim of this paper is to answer the following three research questions:

1. **RQ1:** Is there an improvement gained by hybridizing two algorithms: one from the “search-based” category and the other from the “data mining” category as compared to implementing a single algorithm? How to evaluate the effectiveness of the hybridized algorithm in refactoring sequence diagrams using quality metrics such as cohesion (LCOM2), coupling, recall and precision measures?
2. **RQ2:** To what extent our hybridized algorithm can be effective using another hybridized algorithm and another refactoring operation?
3. **RQ3:** To what extent the search-based algorithm can be effective in a large case study with hundreds of refactoring opportunities?

To the authors’ knowledge, there are no articles that discuss the implementation of search-based or data mining algorithms to refactor sequence diagram models. Hence, the first step is to apply one search-based algorithm to the sequence diagram refactoring problem. The selected algorithm is SA. Then, we hybridize SA with a data mining algorithm which is Kmeans clustering algorithm (KSA). After obtaining the results from the first algorithm, a hybridized algorithm will be run to show the advantages of hybridizing a search-based and data mining algorithm together on a software engineering development problem, namely sequence diagram refactoring. Later, we applied another hybridized algorithm composing of Hill climbing and Kmeans (KHC) for comparison. We also run KSA for another refactoring operation which is Extract Message operation.

This paper is organized as follow: Section 2 presents the background and the previous work in the area of utilizing various algorithms for sequence diagram refactoring; Section 3 discusses the research methodology and the experiment set-up; Section 4 presents the results, the discussion and the threats to validity; and finally, Section 5 contains the conclusion and the future work.

## Related work

Several studies on refactoring UML models have been proposed. Mens et al. [20] acknowledged the usefulness of performing refactoring on higher abstract levels of a software system, such as design levels. Sunye et al. [8] started the research in the UML refactoring domain via their well-known article, Refactoring UML Models. They illustrated refactoring rules on two popular UML diagrams: class and statechart diagrams. As acknowledged in their paper [8], finding refactorings in UML diagrams is not straightforward and further research is required.

Misbhauddin and Alshayeb [21] compared different approaches used in the literature for refactoring UML diagrams. They constructed a criteria-based framework for comparison. The selected approaches are: graph-based, logic- based, direct manipulation, language specific and text-based approach. The following criteria were chosen to compare these approaches: object-oriented concepts, formality, ease of use, conciseness, artifact coverage, expressiveness, granularity, automation, portability and rule handling. Their article provides a holistic view of the merits and drawbacks of each approach for any researchers interested in refactoring UML diagrams.

Misbahuddin and Alshayeb [22] searched the literature on UML refactoring using a systematic literature review and found that only 16% of papers dedicated to UML refactoring discuss sequence diagram refactoring.

Al Dallal [23], in his systematic literature review, illustrated that the most common approach to identify refactoring actions is to utilize quality metrics, with around 32% of all papers applying refactoring operations. Al Dallal also indicated that clustering techniques for refactoring were utilized by around 23% of all surveyed papers. The clustering techniques were mostly based on the similarity between two methods or between a method and attributes. In addition, Al Dallal asserted that cohesion and coupling metrics are the most commonly used in the existing studies to apply quality metrics to evaluate the refactoring process.

Maneerat and Muenchaisri [24] proposed machine learning techniques for bad smells detection of UML diagrams. They applied seven different machine learning algorithms to detect various bad smells such as: lazy class, message chains, middle man, etc. However, they restricted their research to class diagrams only.

Fourati et al. [4] applied quality metrics in order to detect anti-patterns in some UML diagrams, including sequence diagrams. Their work revealed that the cohesion metric, in line with other metrics, can aid in detecting some abnormality in the sequence diagram. They illustrated with examples how quality metrics can unleash four common anti-patterns namely: Blob, Lava Flow, Functional Decomposition and Poltergeists. However, unlike our research, they did not apply any search-based algorithms to detect abnormalities and refactoring to refactor them.

Alkhalid et al. [17, 25, 26] applied clustering techniques at different software levels. They showed that clustering can improve the cohesion and coupling metrics of software code if similarity distance is considered. In their papers, they applied four different clustering algorithms on different open source projects with a fixed and variable number of software entities under study, which are package, class and function. Their research shows that clustering can be very promising in providing refactoring decisions to the user which clearly reflects on the quality. In [17], they applied clustering to a software package and highlighted that these package classes can be considered the entities while the methods are considered the features, thus an entity-feature matrix can be constructed to guide the clustering algorithm. In [25], they applied clustering to software classes and assume that the function should be moved to a certain class based on the number of attributes it accesses. Therefore, the methods are the entities and the class variables are the features. Similarly in [26], they constructed an entity-feature matrix by considering the function statements as entities and their attributes as the features.

Ghannem et al. [5] used an interactive genetic algorithm that prompts and interacts with the designer to help him to refactor a class diagram. Their paper targets a learning-based algorithm where the algorithm learns from a base of examples in order to generate refactoring decisions to the user. However, their data is converted from code to UML diagrams using a tool. Their articulation of the interactive genetic algorithm steps to model refactoring can help researchers to understand how these heuristic algorithms can be applied to refactor UML models.

Amal et al. [19] proposed a hybridized search-based algorithm to solve refactoring problems. Our approach is similar to their approach in the sense that both approaches merge two algorithms: one from data mining and the other from the search-based group. However, there are some substantial differences between this paper and their paper. Amal et al. used a search-based algorithm (Genetic Algorithm) as an input to a machine learning algorithm (Artificial Intelligence Algorithm). Thus, the result of search-based algorithm is used to assist the machine-learning algorithm to perform better. In our case, it is the opposite; we used the machine learning algorithm’s (Kmeans) results to help the search-based algorithm (SA) to perform better. Furthermore, our approach is fully automated without intervention or interacting with the designer during the run of the algorithm. In their paper, after a few runs of the GA, the designer evaluates the results manually before feeding them to the ANN algorithm. In addition, they applied their approach at the code level, while our approach is targeting the sequence diagram on the design level. Although, we both used recall and precision to evaluate our algorithm, our approach takes it one step further by ensuring that any recommended refactoring must improve two competing software metrics (coupling and cohesion).

In addition to these approaches, representing the refactoring problem as a multi-objective method has been discussed and implemented for software code, model refactoring and maintainability [27–30].

The above approaches suffer from drawbacks, which motivated us to consider the utility of hybridization. Evolutionary computing methods are known to be computing-intensive [31] due to the population size and the number of generations required to converge. In addition, there are many parameters such as crossover operation, mutation operation, and selection operation etc. that should be set correctly in order for the algorithm to produce promising results.

Clustering is very effective for optimizing cohesion and coupling simultaneously but it is a computation-intensive algorithm for large data [32]. Search-based algorithms can work only on one objective, for more than one objective, such as coupling and cohesion considered in this paper; a multi-objective version should be considered which adds more to the computational demand. Thus, combining clustering with a simple search-based algorithm such as SA could produce good results. SA has two parameters that can be easily set. In [14], only one parameter was shown to have a great impact which is the cooling factor. Thus, it motivated us to apply this hybridization for a sequence diagram refactoring. Table 1 summarizes the surveyed approaches and compares them with our proposed approach.

**Table 1:** Summary of literature review.

Author and Years	Methodology Approach	Artificial Intelligence Algorithm	Detection / Refactoring	Refactoring Level	Evaluation Method
Alkhalid et al. 2011, 2010, 2011, [17, 25, 26]	Deterministic	Clustering	Code detection and refactoring	Function Class Package	Coupling and cohesion metric
Ghannem et al. 2013, [5]	Search-Based	Interactive Genetic Algorithm	Class model detection and refactoring	Method & Class	Recall and Precision
Maneerat and Muenchaisri, 2011, [24]	Deterministic	-	Class model detection	Method & Class	Accuracy, Specificity, sensitivity,. etc.
Sahin et al. 2014, [27]	Search-Based	Genetic Programming and Genetic Algorithm	Code detection and refactoring	Method & Class	Recall and Precision
Ouni et al. 2013, [28]	Search-Based	Non-Dominated Sorting Genetic Algorithm–II.	Code detection and refactoring	Method & Class	Recall and Precision
Kessentini et al. 20111, [13]	Search-Based	Genetic Programming	Code detection and refactoring	Method & Class	Recall and Precision
Amal et al. 2014, [19]	Hybridization of Deterministic and Search-Based	Neural Network and Genetic Algorithm	Code detection and refactoring	Method & Class	Recall, Precision and Refactoring Efficiency
The proposed approach	Hybridization of Deterministic and Search-Based	Kmeans and SA	Sequence diagram detection and refactoring	Method & Class	Cohesion, Coupling, Recall and Precision

## Research methodology

In this paper, we use a single and hybridized algorithm to perform refactoring on sequence diagrams. SA is a search-based algorithm candidate for sequence refactoring. Two hybridized algorithms are applied namely: KSA as an abbreviation of the combination of Kmeans and SA [33] and KHC as an abbreviation of the combination of Kmeans and HC.

**Fig 2:**
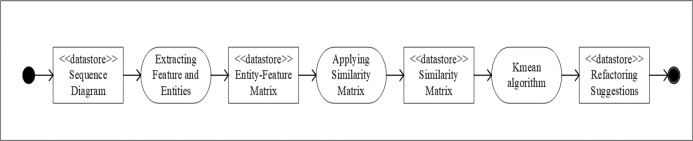
Kmeans algorithm steps applied for sequence diagram refactoring.

To illustrate how our KSA algorithm works, we explain the process of each algorithm separately and then we explain the hybridized process. Fig 2 shows the steps for the Kmeans algorithm to refactor a sequence diagram. As illustrated in Fig 2, there are two major preprocessing steps required for this algorithm, which are: extracting entities and features and constructing a similarity matrix. The extraction of entities and features sub-process is explained in detail in subsection 3.4, while the construction of the similarity matrix is explained in detail in subsection 3.5. After these two sub-processes, the Kmeans algorithm is run, as detailed in subsection 3.1.

**Fig 3:**
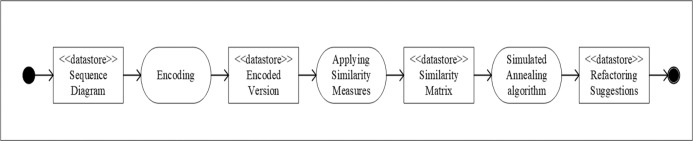
SA algorithm steps applied to sequence diagram refactoring.

Fig 3 shows the steps for the SA algorithm to refactor the sequence diagram. The sequence diagram should be converted into a medium representation. Since our objective is to hybridize SA with Kmeans, we used the same representation for both of them.

**Fig 4:**
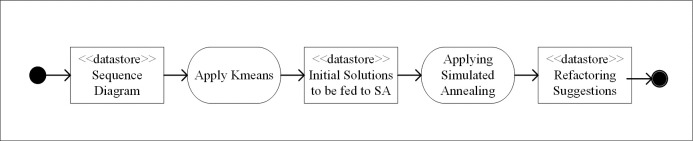
KSA algorithm steps applied to sequence diagram refactoring.

Fig 4 shows the steps of the proposed KSA algorithm. The details of this algorithm are explained in detail below.

In the first step, the sequence diagram is converted into an entity-feature matrix. After this, the data mining algorithm (Kmeans) will use the constructed entity-feature matrix to create clusters. Each cluster groups the most similar objects together. Therefore, in this paper, each cluster contains messages that have similar features.

In the next step, we have a collection of clusters where each cluster groups the most similar messages together. This is the initial solution on which the second algorithm will run. The search-based algorithm (SA) will start from within the clusters returned by the Kmeans algorithm instead of starting randomly from any position. SA will be guided by metrics to refactor the sequence diagram. The refactoring operations will be “moving messages”, and “extracting message”. The algorithm will apply the sequence of this refactoring operation on random points of the cluster returned by the Kmeans algorithm.

Finally, the hybridized algorithm will provide suggestions on sequence diagram refactoring using the “move message” and “extract message” operations.

### Kmeans clustering

Clustering is considered as one type of data mining and machine learning process [34]. Its popularity is attributed to the increase of interest in the Internet and the huge demand to analyze large Internet data. Clustering is a process that collects similar data objects in one group and dissimilar ones in other groups [34]. Clustering algorithms have no idea or guidance on the objects in advance and iteratively try to collect features from these objects and divide them into various groups called clusters. Unlike metaheuristics, where problem types play a major role in determining the difficulty of the problem to be solved by a certain metaheuristic, in clustering, data types contribute the most to clustering algorithms. This observation is obvious since metaheuristics collect information from the problem or solution space to guide the search, where in clustering techniques, the objects data (attributes, features, types, etc.) guide the clustering algorithm. Although there are numerous clustering algorithms in the literature [35, 36], many of them are based on similarity measures.

**Fig 5:**
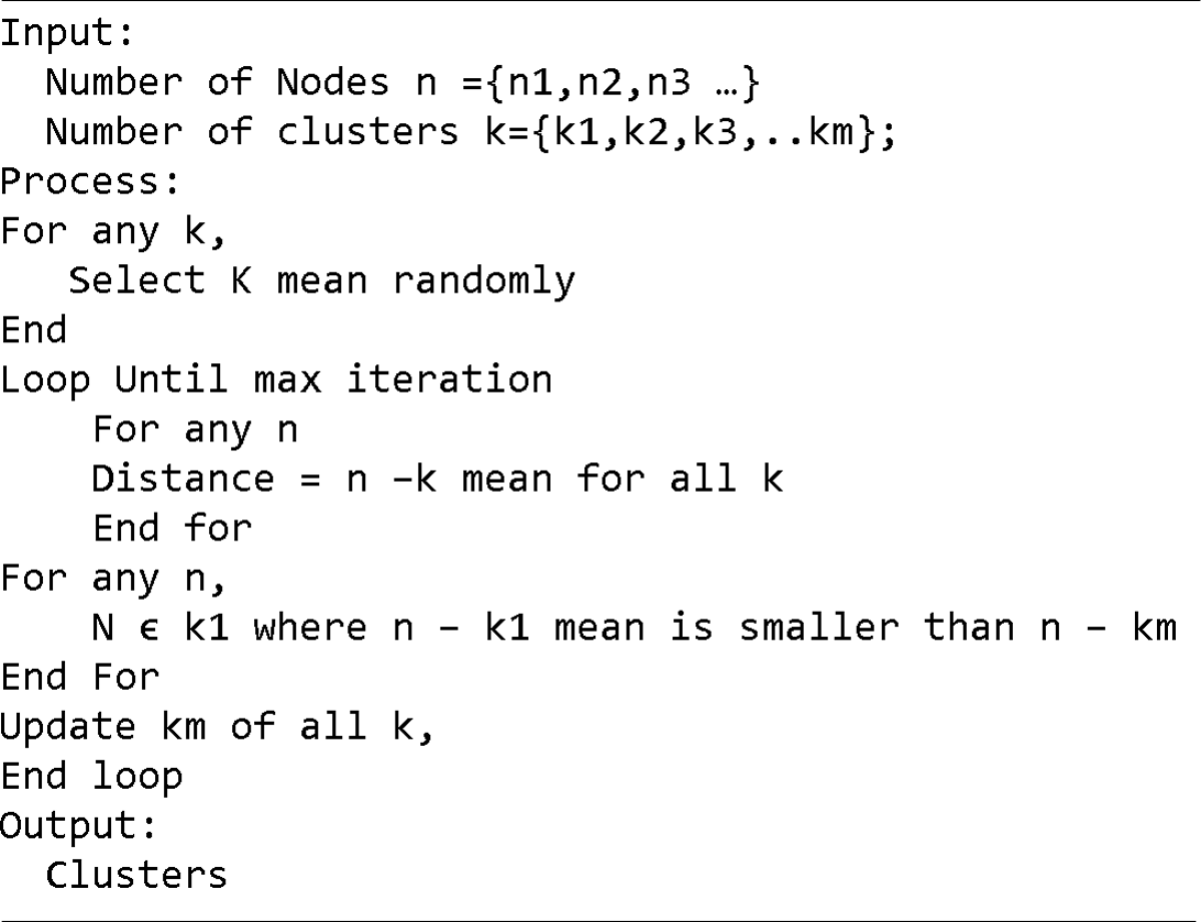
Kmeans algorithm pseudo code.

Kmeans is a simple clustering algorithm used to group objects together based on their distance [34]. At the beginning of the algorithm, the cluster mean is initialized randomly and it is updated in each iteration. The algorithm continues to gather objects into clusters and updates the mean until a stopping condition is met or the cluster mean is not updated. Fig 5 shows the pseudo code of the Kmeans algorithm.

### Simulated annealing

**Fig 6:**
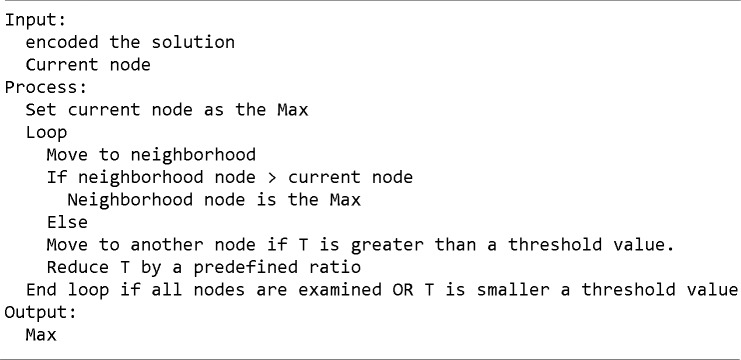
SA algorithm pseudo code.

SA is an optimization algorithm inspired from physics [33]. It has several applications in science and engineering due to its simplicity to implement and its few parameters such as: temperature, acceptance probability and the cooling factor. The algorithm starts with a predefined temperature (T) set by the user. It starts from a random point and calculates the objective function. In each iteration, the algorithm moves to a neighbor point and calculates its objective function. If the neighbor point has a better value using the objective function, then the algorithm will select it and move to it. If the value of the neighbor point using the objective function is lower, then it will accept it with a pre-defined acceptance probability (P). The algorithm will repeat the same process, decreasing T using another parameter called a cooling factor (CF). If the CF is large, this is called fast annealing. If the CF is small, this is called slow annealing. The determination of CF along with the acceptance probability (P) is critical for the algorithm to return better results. Fig 6 shows the pseudo code of the SA algorithm.

### The proposed solution

There are several general limitations in Kmeans clustering algorithms: 1) the Kmeans algorithm tends to get stuck at a local optimum point; 2) the number of clusters must be specified in advance; 3) the random initialization of cluster means can be far from all points, and 4) the Euclidian distance is based on points in two-dimensional planes, and the entity-feature matrix must be constructed. These limitations are also applicable to problems in software engineering domain.

Likewise, SA has several limitations similar to other search-based algorithms: 1) the SA algorithm tends to get stuck at a local optimum point; 2) the first initial number might be far from the optimal solution; 3) it requires a fitness function; and 4) it has some controlling parameters such as temperature and probability that must be tuned.

The KSA hybrid algorithm seeks to optimize the performance of the SA algorithm by providing better initial points of SA using the Kmeans algorithm. The Kmeans algorithm will run first on the problem where it will extract the points that have a potential improving value and gather them into one cluster. This cluster then will be passed to the SA algorithm to start randomly from any position within this cluster. This will improve the performance of the SA algorithm by letting it start from a promising point in the search space. Then, at each iteration, the SA algorithm will move to another point within this passing cluster in order to ensure that the SA algorithm proceeds from one promising point to another promising point and reduce the chance of being trapped in local optima. In addition, this communication between the Kmeans algorithm and the SA will save many iterations where SA will investigate weak points that will slow the algorithm convergence.

Two experiments have been performed to show the advantages of KSA over SA. Experiment 1A shows the performance using SA alone, while experiment 1B shows the performance of KSA. KSA does not solve the limitation of Kmeans. Kmeans can still get stuck into local optima. In the literature, several papers addressed how SA can be used for optimal selection of initial Kmeans points [37, 38]. The output of these results is that metaheuristics can solve the initialization problem of Kmeans, but the problem of converging into local optima is still not solved. On the other hand, other papers show the advantage of Kmeans in reducing the computation time of SA [39]. The clustering algorithm (Kmeans) will take all the points (messages) and cluster them based on their high similarities with the corresponding classes. Then, the SA algorithm will save time by picking points that have the potential to reflect on the sequence diagram refactoring.

The hybridized KSA algorithm starts by providing k cluster centers randomly. Then the algorithm continues updating the centers of the clusters until all points are assigned to one cluster; that cluster includes all points that might make a contribution to the problem under study which is the degree of similarity between two components of sequence diagrams. The KSA will pick points out of this cluster to ensure that any point taken should have a similarity value; thus, it improves our designed cost function. This does not imply that Kmeans will always present the optimal points to SA since as we stated earlier, Kmeans might converge into local optimum points. Still, even though Kmeans might not provide us with the best SA initial points, it can help in providing some of the relative points that assists SA into reducing its cost function faster.

**Fig 7:**
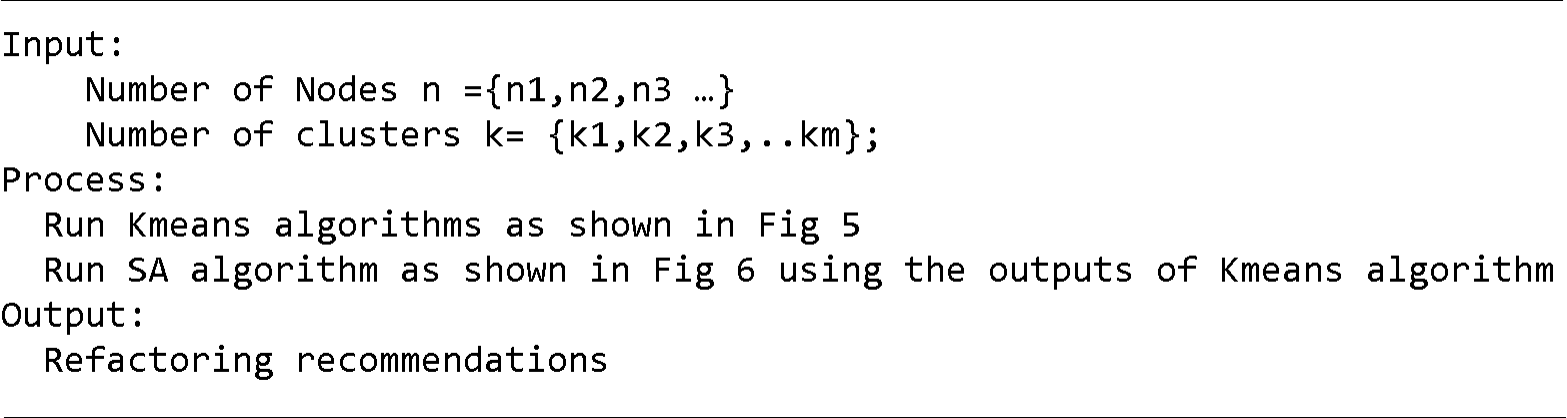
KSA hybridized algorithm pseudo code.

In short, we are considering three cases: the first case is that Kmeans can provide optimal points to SA if it does not converge into local optima. The second case is: when Kmeans converges into local optima after a few loop iterations, the returned points of Kmeans can still guide SA. The last case is running SA alone without Kmeans. To solve our problem, the first two cases are better than the third case as the search space of the problem contains many irrelevant points that do not contribute to the cost function. For example, in experiment 3, we have 10000 messages and 100 classes. However, there are only 1000 anti-pattern instances; in other words; only 1000 points out of this huge search space can contribute to the cost function. Therefore, KSA is more appreciated than SA alone in this situation. Fig 7 shows the pseudo code of the KSA hybridized algorithm.

### Entities and features

Clustering algorithms depend on grouping entities together, based on the similarity value found in their features. It is important to select a number of features that reflect the similarity between entities. Selecting too many features may result in clustering each entity in a separate group. Likewise, selecting too few features may end up with crowding a few clusters with many entities that do not relate to each other. Hence, the selection of features should be considered when designing clustering algorithms to solve a particular problem.

Entities are the objects that we want to cluster. In our proposed solution, a sequence diagram can be treated as a set of entities with different features. Our objective is to refactor sequence diagrams using a hybridized algorithm and evaluating the results using selected quality metrics. To achieve this, we are going to group similar messages in the most suitable class. For sequence diagram refactoring, methods are considered entities. Each message has two features 1) the name of the function it is sending to or receiving from; 2) message type (a direct or an iterative message). In the entity-feature matrix, we record how many features of the message each class shares, which can be none, one or more features. In our case study, we used the method parameters as the message features as explained in section 3.5. However, our algorithm works on any other representations of features.

In a sequence diagram, cohesion can be defined as the number of messages a class is able to access within itself. The highest cohesion is required since it will reduce the number of communication messages with other classes. There are different metrics for measuring cohesion. We selected LCOM2 [40] as the cohesion metric as in [25] for comparison purposes and for its applicability to our proposed solution that is based on similarity. LCOM2 is found to be more suitable for our study because it considers calculating the shared attributes between methods; other cohesion metrics are found to be less relevant. For example, LCOM3 and LCOM4 do not consider the number of attributes that are shared between two methods [41]. Loose Class Cohesion (LCC) represents the connection between public methods without considering the sharing of instance variables, so similarity between methods are not considered which rendered the usefulness of clustering algorithm.

We define coupling as the number of direct messages the class is sending to or receiving from other classes. Reducing the coupling value is desirable due to the fact that it will reduce the communication messages between classes.

A higher similarity of features indicates similarity of functionality, which in turn increases cohesion and reduces the coupling of message communication between different classes. The advantage of such processes is to increase the quality of the model, as cohesion and coupling are desirable features in object-oriented software systems.

### Similarity matrix

A similarity matrix is a matrix where rows represent features and columns represent entities. The value inside each matrix cell represents how many features there are in one entity. The application of the Entity-Feature matrix for software refactoring was originally proposed by Lung et al. [42].

Since clustering algorithms are based on similarity distance, clustering algorithms are applied for software refactoring to enhance cohesion and coupling. Cohesion implies that if a class has many similar features of a particular method, then that method should be a member of that class. Thus, by knowing which class has the most similarity with a method, we can move that method to that class. Similarly, coupling refers to the communication between two different entities. By knowing which method should belong to which class, we can reduce the number of communication messages between these entities. Software developers aim to increase the cohesion of the software and minimize its coupling.

Table 2 is adopted with some modifications from Alkhalid et al. [25] to compare with the authors’ results. In their paper, Alkhalid et al. applied only clustering algorithms to refactor software classes. In this paper, we use a hybridized algorithm. We use this table for comparison purposes and due to the scarcity of sequence diagram data that is representative enough in order to show the benefits of the proposed approach.

**Table 2:** Similarity matrix between entities and features.

	Class 1	Class 2	Class 3	Class 4	Array Index
Message1	1	2	0	0	0–3
Message2	2	0	0	0	4–7
Message3	2	0	0	0	8–11
Message4	2	0	0	0	12–15
Message5	0	2	0	0	16–19
Message6	0	0	1	2	20–23
Message7	1	2	0	0	24–27
Message8	0	0	0	2	28–31
Message9	0	0	2	0	32–35
Message10	0	0	2	0	36–39
Message11	0	0	2	0	40–43
Message12	0	0	2	0	44–47
Message13	2	1	0	0	48–51
Message14	1	0	2	0	52–55
Message15	0	0	0	2	56–59
Message16	0	0	0	2	60–63

**Fig 8:**
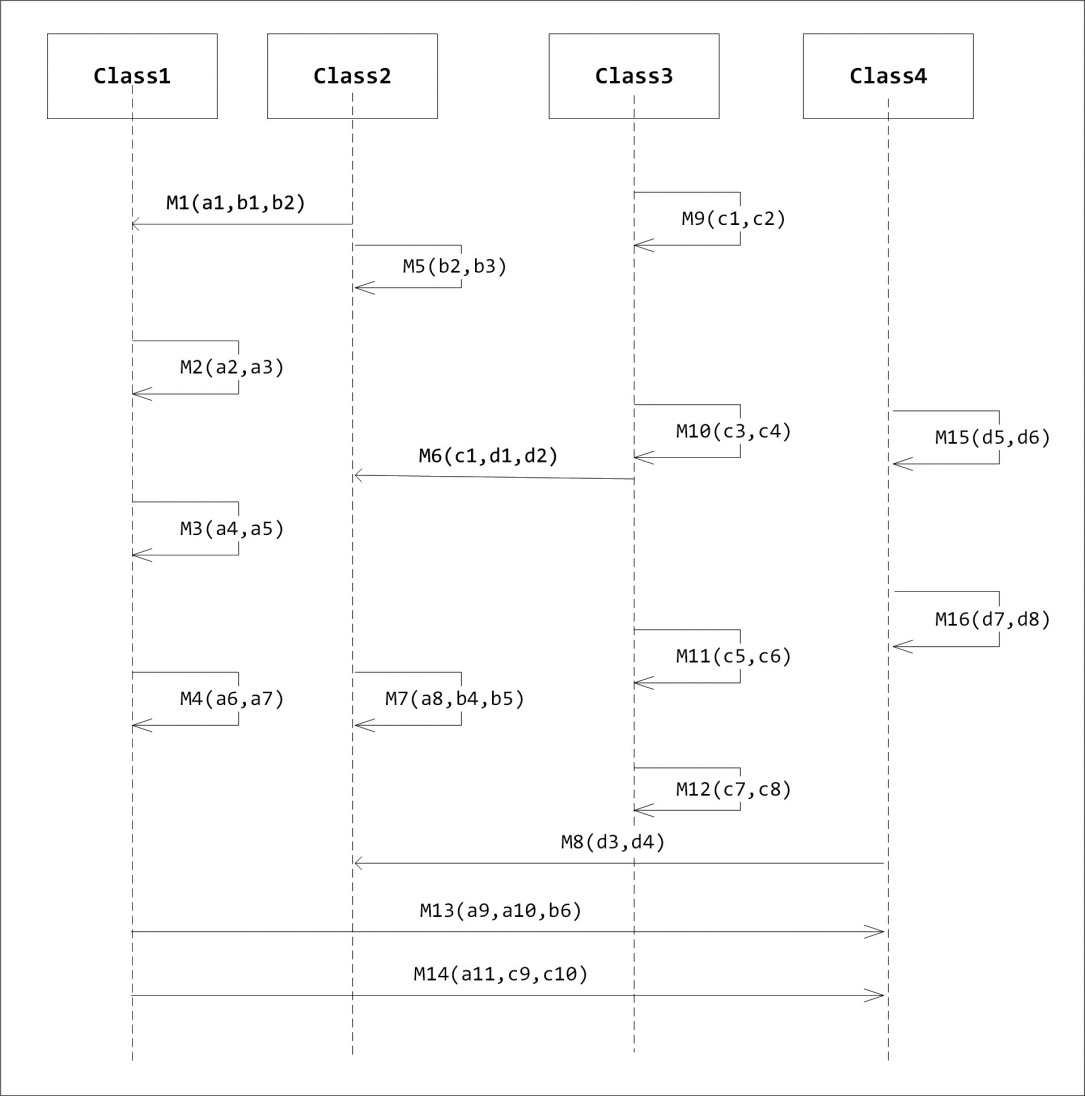
Sequence diagram case study.

Fig 8 shows the sequence diagram example represented by Table 2. As we can see, we have four classes and sixteen messages. Variables belong to class1 are named by the letter ‘a’ followed by a sequence number. Likewise, variables of class2, class3 and class4 are named by the ‘b’, ‘c’ and ‘d’ respectively followed by a sequence number. Each message contains parameters. These parameters are the features that we are looking for in order to apply the algorithm. For instance, Message 1 denoted by M1 in the diagram representing a message that is sent from class2 to class1 and it has three parameters: a1, b1, and b2. The parameter variable a1 belongs to class1 and the parameter variables b1 and b2 belong to class2. Message2 represents a method that calls itself and thus in this case it has only the variables named with ‘a’.

Table 2 shows the entities and features of a sequence diagram case study. Value 1 in the intersection of the Class 1 column and Message 1 row indicates that class 1 has only one feature of message 1 while class 2 has two features of the same message. Class 3 and class 4 do not share any features with this message. Thus, to increase cohesion, the algorithm will propose moving message 1 to class 2. We implemented this table internally using array. Table 2 has 64 values and thus it was represented by an array that starts from 0 and ends with 63.

Table 3 shows the self-messages in each class. This is similar to the adopted case study. However, in the adopted case study, the relationships are between classes and methods. In this paper, we modify the relations to be between classes and messages. Therefore, we have 4 classes and 16 messages where each class has self or communicating messages. Tables 4 and 5 show the initial cohesion and coupling values of each class respectively, while Table 6 indicates the necessary refactoring operations in order to reach an optimal state in terms of cohesion and coupling metric values.

**Table 3:** Classes and belonging messages.

Class	Self-messages
1	2, 3 and 4
2	5 and 7
3	9, 10, 11 and 12
4	15 and 16

**Table 4:** The cohesion value of the classes in our case study.

Class	Relations between messages	Cohesion Value
Class 1	M1 ∩ M2, M1 ∩ M3, M1 ∩ M4, M2 ∩ M3, M2 ∩ M4, M3 ∩ M4	Mn = 2, Ms = 3 LCOM = Max (2–3,0) = 0
Class 2	M5 ∩ M6, M5 ∩ M7, M5 ∩ M8, M6 ∩ M7, M6 ∩ M8, M7 ∩ M8	Mn = 3, Ms = 3 LCOM = Max (3–3,0) = 0
Class 3	M9 ∩ M10, M9 ∩ M11, M9 ∩ M12, M10 ∩ M11, M10 ∩ M12, M11 ∩ M12	Mn = 1, Ms = 5 LCOM = Max (1–5, 0) = 0
Class 4	M13 ∩ M14, M13 ∩ M15, M13 ∩ M16, M14 ∩ M15, M14 ∩ M16, M15 ∩ M16	Mn = 4, Ms = 1 LCOM = Max (4–1, 0) = 3

**Table 5:** The coupling values of the classes in our case study.

Class	Coupling
Class 1	1
Class 2	2
Class 3	0
Class 4	1

**Table 6:** The required refactoring operation in each class to reach an optimal cohesion and coupling state.

Class	Required Refactoring operation
Class 1	1
Class 2	1
Class 3	1
Class 4	2

The cohesion values are generated using the LCOM2 metric. To calculate the LCOM2 of a class: first, count the number of messages that share features, denoted as (Ms). Then, calculate the number of messages that do not have any features in common, denoted as (Mn). If (Mn)—(Ms) > 0, the LCOM2 is (Mn–Ms). Otherwise, LCOM2 is zero. Therefore, LCOM2 can be non-negative. Table 4 shows the value of cohesion for each class based on the LCOM2 metric.

Table 5 shows the coupling values of the sequence diagram before refactoring. In this paper, coupling refers to the coupling between classes. It indicates the number of messages communicated between two classes. For instance, Class 1 has a coupling value of 1 because it has only one message (Message 1) that is communicated to another class (which in this case is class 2).

Table 6 shows the number of refactoring operations that are required to move the system into an optimal state in terms of cohesion and coupling. In this paper, we use the “move message” operation, which is similar to the “move method” operation used by Alkhalid [25]. As shown in Table 6, though classes 1, 2 and 3 already have an optimal LCOM2 value of zero, they are still involved in the refactoring process. Applying refactoring on class 4 only might reduce the LCOM2 of class 4, but increase the coupling or even the LCOM2 of the other classes. Thus, balancing both metrics is essential.

## Results & discussion

In this section, we show the results of our two experiments: one using the SA algorithm and the second using our hybridized KSA algorithm. The experiment was carried out using an Intel-based computer powered by a 3.30 GHz processor and 4 GB memory. The code was implemented on a Windows 7 system using Java. The parameter setting of both algorithms is:

Kmeans has two parameters: the number of clusters and the randomized function for cluster’s center. Since we have four classes in our case study, we set the number of clusters to be four; the center of each cluster is determined randomly using the following function:

$$C_{k}=1+9.00\;*\;\text{rand}.\text{nextDouble}();\;\text{where}\;C\;\text{represents the center and}\;k\;\in \{1,4\}$$ (1)

SA has two parameters to be set: initial temperature and the Cooling Factor. The cooling factor is fixed in all experiments, but the initial temperature is changed. The parameter settings of SA are shown in the result sections. These parameters are set arbitrarily using trial and error until this setting found to return good results.

The objective of this experiment is to answer RQ1 “Is there an improvement gained by hybridizing two algorithms: one from the “search-based” category and the other from the “data mining” category as compared to implementing a single algorithm?” How to evaluate the effectiveness of the hybridized algorithm in refactoring sequence diagrams using quality metrics such as cohesion (LCOM2), coupling, recall and precision measures?

### Experiment 1.A: Refactoring sequence diagram model using SA

We implemented an SA algorithm on the above case study to provide suggestions to the users on how to move messages between the different classes in order to maximize cohesion. This is a demonstration experiment to show the limitation of the SA algorithm. However, when we run our hybridized algorithm, the four selected metrics are discussed. The SA algorithm is based on a random initialization of one point. The algorithm can randomly pick any point out of the 64 points shown in Table 2. After this, the algorithm determines the class to which the selected message should belong. Then, based on similarity features, the algorithm will recommend either moving the message to another suitable class or recommend to keep the selected method in the same class. After all the recommendations of the SA algorithm, the LCOM2 value is calculated. LCOM2 measures lack of cohesion, hence reducing the value of this metric is desirable. Therefore, in the above example, Class 4 has an LCOM2 value of 3 which needs to be reduced.

We ran SA algorithms 10, running the SA algorithm returns this sequence of random numbers (57, 55, 37, 38, 25, 2). The first number is 57, which corresponds to the array index 57 in Table 2. Here, 57 corresponds particularly to the number of features that “Message15” shares with “Class2”. As shown in the table, there is no common feature between “Class2” and “Message15” and subsequently, picking up this point will make no contribution to reducing cohesion or increasing coupling. In this case, the SA algorithm will pick another point randomly if the probability threshold is not met yet.

The next point the algorithm selects is 55. Again, 55 corresponds to the number of features in common between “Class4” and “Message14” which in this case is null. Therefore, picking up this point will not improve cohesion or coupling and the algorithm continues to pick another point as long as the probability threshold is still satisfied. The third point is 37, which also does not have any impact on the cohesion or coupling value. The same applies to all remaining points.

In another run, the following sequence of numbers was generated (3, 61, 40, 23, 29, 2). In this sequence, point 23 corresponds to the number of features in common between “Message6” and “Class4”. According to Table 2, Message6 initially belongs to “Class2”, though it is more similar to “Class4”. Therefore, the SA will recommend moving “Message6” to “Class4”. Table 7 shows the results of running SA algorithm using the case study. The algorithm, in 7 iterations, was unable to find any refactoring that reduces cohesion. Thus, the LCOM values remain the same. LCOM values are unchanged by running SA algorithm on this case study since the algorithm failed to provide any recommendation.

**Table 7:** Results of the first run of SA algorithm.

No.	Point	KSA recommendation			
1	47	Keep it			
2	37	Keep it			
3	31	Keep it			
4	39	Keep it			
5	48	Keep it			
6	1	Keep it			
7	47	Keep it			
LCOM		0	0	0	3
Initial Temperature		10000000.0			
Running Time		4.5 seconds			

#### Limitation of this approach

The algorithm will pick any point randomly. The algorithm might start from a very bad point such as the “0” point in Table 2. This will affect the algorithm’s performance since the algorithm might waste some iterations picking up the “0” value which is not useful because it indicates no common feature of a message in a class. In addition, the existence of these bad points might converge the algorithm quickly to local optima. Consider the situation where the algorithm picks this sequence of values randomly (2, 0, 0, 0, 0, 0, 0, 0, 0, 0). In this sequence, the algorithm starts from a good point where it has a maximum similarity of features, but it keeps going to one weak neighbor to another. After a few iterations, the acceptance probability will be low and the algorithm will hit the condition criteria, forcing it to terminate.

### Experiment 1.B: Refactoring sequence diagram model using a hybridized SA and clustering algorithm

We propose the hybridized SA algorithm and the Kmeans using a pipeline fashion [43]. Pipeline hybridization means that algorithm “A” runs fully and its results are taken to algorithm B as inputs. This type of hybridization has been investigated in several papers concerning Kmeans and SA algorithms [18, 44, 45]. The clustering algorithm (Kmeans) will take all the points (messages) and cluster them based on their high similarities with the corresponding classes. Then, the SA algorithm will save time by picking points that have the potential to reflect on the sequence diagram refactoring.

When we run our hybridized KSA algorithm, the following sequence of points appear (38, 49, 4, 25, 1, 1, 59). This is the final results of points picked up by our hybridized algorithm. Before we delve more deeply into analyzing these results, let us look at the intermediate results. The KSA algorithm starts by providing four cluster centers randomly since we have four classes. These centers are at the points {4.3, 6.3, 9.3, 3.9}. Then the algorithm continues updating the centers of the clusters until all points are assigned to one cluster which includes all points that might make a contribution to the cohesion metric, that is, the points that indicate that there are similar features between messages and classes. Now, the KSA will pick up points out of this cluster to ensure that any point taken should have a similarity value and hence it might, but not necessarily, reduce cohesion or increase coupling.

If we check all the points in this sequence as returned by KSA: (38, 49, 4, 25, 1, 1, 59), we find that all these points have similarity values. Unlike running SA alone where SA might go for many iterations picking up non relevant points (points with no similar features values), KSA only picks the right point, thus increasing the speed of the algorithm. Table 8 shows the points, the representation of the points and the recommendations by the KSA algorithm. LCOM values of class 3 are reduced by one as a result of the suggested recommendation of moving Message 13 to Class 1.

**Table 8:** Results of the first run of KSA algorithm.

No.	Point	Representation				KSA recommendation	
1	38	Message10, Class3				Keep it	
2	49	Message13, Class2				Move Message13 to Class1	
3	4	Message2, Class1				Keep it	
4	25	Message7, Class2				Keep it.	
5	1	Message1, Class1				Keep it	
6	1	Message1, Class1				Keep it	
7	59	Message15, Class4				Keep it	
Metric /Class		1	2	3	4	Recall	20%
LCOM2		0	0	0	2	Precision	20%
Coupling		1	1	1	1	Ratio	7/ 1 = 7
Initial Temperature of SA		10000000.0		Termination Condition	Temperature > 1	Cooling Factor	Temperature /10.0
Running Time		3 seconds					

The following details show the answer of RQ1 “How to evaluate the effectiveness of the hybrid algorithm in refactoring sequence diagrams using quality metrics such as cohesion (LCOM2), coupling, recall and precision measures?”

Table 8 shows the values of LCOM2, coupling, recall, precision and the ratio. Moving message 13 to class 1 will reduce the cohesion of class 4 to 2 where its initial value is 3, as shown in Table 4. In addition, the coupling of class 2 is reduced to 1 where its initial value is 2, as indicated in Table 5; however, the value of LCOM2 for class 1 is still 0. This is an optimal value of cohesion and moving the message leaves it in this state. Meanwhile, the KSA algorithm recommends one refactoring operation out of 5 required operations to lead the system into an optimal state. So the recall is 1/5 or 20%. The recommendations provided by the algorithm are correct, so again the precision is 1 correct refactoring operation out of 5 which is 20%. The ratio indicates how many iterations result in the refactoring operation in comparison to the number of iterations the algorithm undertakes. In this run, the algorithm runs for seven (7) iterations where only one iteration recommends a refactoring operation. So the ratio is 7. The ratio can tell us how good the algorithm is for finding the good results. A ratio of 7 is not considered a good value since the algorithm has to waste six other iterations to find one good point in one iteration.

All recommendations of KSA are correct and whether it recommends moving messages or leaving them in their original class, the algorithm is able to determine the class for each message. Our case study involves four classes with three classes have a good cohesion value and the fourth having a high cohesion value. This is a difficult scenario for the algorithm since picking 75% of space points will not result in a good value. Thus, we run another experiment by relaxing the probability threshold to allow the algorithm to run for a few more iterations.

In the second run, as shown in Table 9, we relaxed the acceptance probability so the algorithm can continue for more iterations than the first run before it terminates. In this run, the algorithm picked 11 random points recommending 4 refactoring operations. So the recall of the algorithm in this run is 4 out of 5 or 80%. All the recommended refactoring operations by the algorithm are correct, so the precision is 80% too. The ratio is considerably good; the algorithm runs 11 iterations to find 4 refactoring operations, which means the ratio is 2.75. This is far better than the first run. We did not calculate coupling and cohesion in this run until all necessary refactoring operations are recommended by the algorithm.

**Table 9:** Results of the second run of KSA algorithm.

No.	Point	KSA recommendation	
1	4	Keep it	
2	12	Keep it	
3	46	Keep it	
4	0	Move Message1 to Class2	
5	31	Move Message8 to Class4	
6	8	Keep it	
7	22	Move Message6 to Class4	
8	23	Keep it	
9	17	Keep it	
10	49	Move Message13 to Class1	
11	22	Keep it	
Recall			80%
Precision			80%
Ratio			11/ 4 = 2.75
Initial Temperature			100000000000.0
Running Time			4.5 seconds

**Fig 9:**
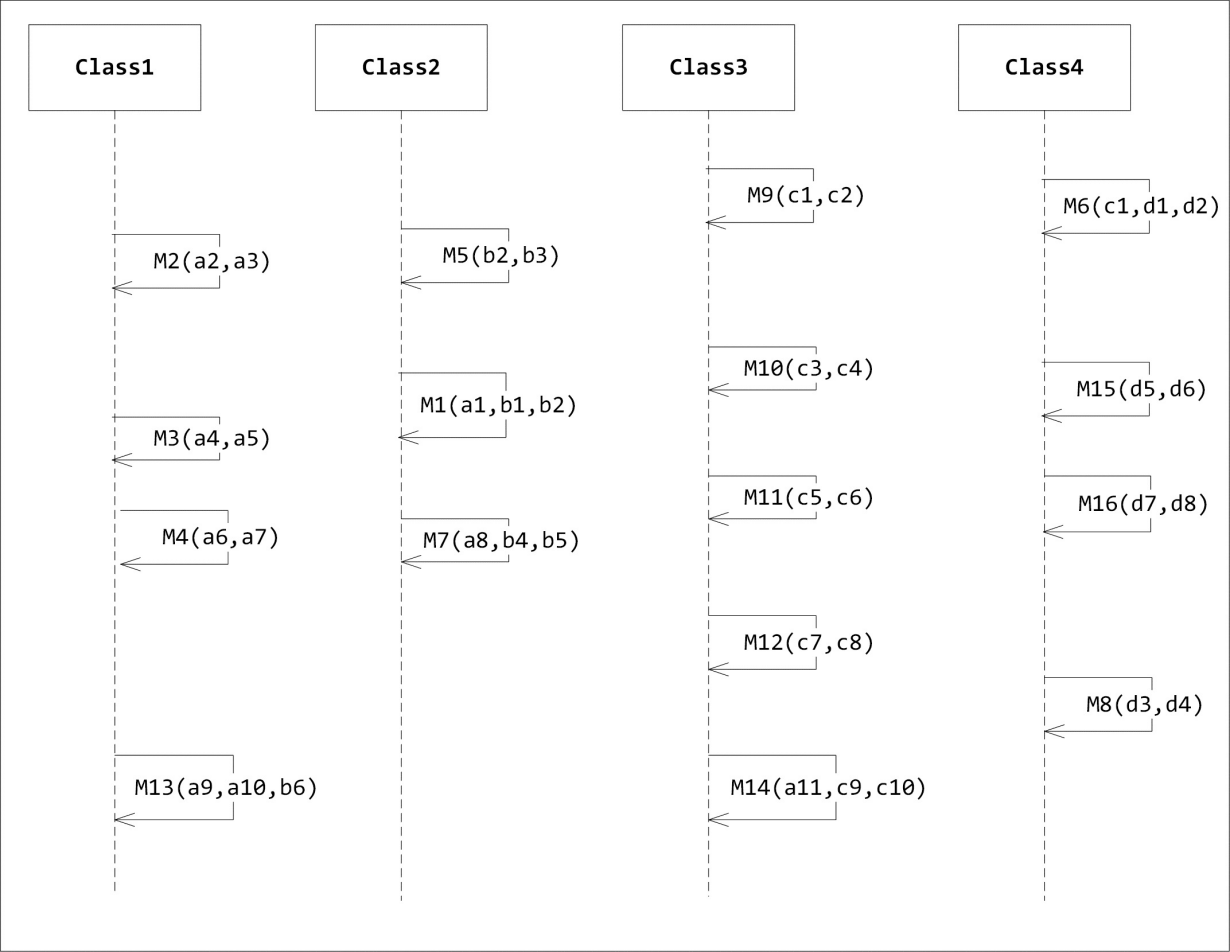
The refactored sequence diagram as recommended by KSA algorithm.

In the third run, as shown in Table 10, the KSA algorithm was able to recommend all necessary operations in order to reach the optimal state, but it has to run for 21 iterations. The LCOM2 and coupling values are now optimized. The LCOM2 value of class4 now is 0 and the coupling of the four classes are 0 too. When the LCOM2 value is decreased, it indicates better cohesion. The value of the coupling metric decreases as well which is a desirable result too. The recall value in this experiment is 5 out of 5 which is 100%. All of these recommendations are correct, so the precision is 100%. However, the ratio is 4.2 since the algorithm takes 21 iterations to find 5 correct operations. This means that the algorithm, on average, has to go for 4 iterations to find one good refactoring. Fig 9 shows the refactored system as recommended by our algorithm.

**Table 10:** Results of the third run of KSA algorithm.

No.	Point				KSA recommendation	
1	4				Keep it	
2	12				Keep it	
3	46				Keep it	
4	0				Move Message1 to Class2	
5	31				Move Message8 to Class4	
6	8				Keep it	
7	22				Move Message6 to Class4	
8	23				Keep it	
9	17				Keep it	
10	49				Move Message13 to Class1	
11	22				Keep it	
12	34				Keep it	
13	49				Keep it	
14	38				Keep it	
15	54				Move Message14 to Class3	
16	38				Keep it	
17	12				Keep it	
18	31				Keep it	
19	23				Keep it	
20	48				Keep it	
21	1				Keep it	
Metric /Class	1	2	3	4	Recall	100%
LCOM2	0	0	0	0	Precision	100%
Coupling	0	0	0	0	Ratio	21 / 5 = 4.2
Initial Temperature	100000000000.0					
Running Time	5 seconds					

In comparison to [25], where they applied only clustering and class4’s LCOM2 was decreased by 2, in our experiment, class4 was decreased by 3. Moreover, they used the Coupling Through Abstract Data Type (CTA) as a coupling metric. The CTA value for class1 is increased by 1, for class2 it is decreased by 1, there is no change for class3 and a decrease by 3 for class4. In this paper, we opt to select a coupling between the messages’ metric due to the difficulty of applying CTA in sequence diagrams. The coupling metric in our paper was decreased by 1 for all four classes.

The purpose of Experiment 2.A and 2.B is to answer RQ2: To what extent our hybridized algorithm can be effective using another hybridized algorithm and another refactoring operation?

In order to provide a fair comparison between the running time of these two algorithms, we opted to compare the SA algorithm against the (SA) code section of the KSA. KSA is a hybridization of two algorithms and in most cases, it is expected to take a longer time doing the clustering part. However, comparing the running time of the SA section of the KSA against the running time of the standalone SA algorithm is in line with our research objective. SA will have to randomly pick a point out of the full set of the available points, while the SA section in the KSA will pick a point out of the output of Kmeans. When the SA succeeds in finding an optimized value in one iteration, it will start a new iteration immediately. However, when it fails, few lines must be executed before starting a new iteration such as: checking the stopping criteria threshold and updating the temperature parameter. Thus, in the KSA, we expect the SA section of this algorithm to run faster than the standalone SA; this is because the probability of SA to fail in finding good points is smaller in the KSA than in the standalone SA. Since this is a randomized algorithm, we ran the algorithm 5 times and took the average.

**RQ2:** To what extent our hybridized algorithm can be effective using another hybridized algorithm and another refactoring operation?

### Experiment 2.A: Comparison with KHC

Table 11 shows the results returned by running a hybridized algorithm of Kmeans and Hill Climbing (HC) algorithms. We run the experiment eight times and recorded the results. The table shows the experiment number, the picked random point and the algorithm’s recommendation. In the first five experiments, the algorithm does not find any refactoring opportunity and that is why it terminates after one run. In the sixth experiment, the algorithm finds two refactoring opportunities. The first refactoring opportunity reduces the cohesion and thus allows the algorithm to go for iteration. Though the second iteration results in recommending a refactoring opportunity but since it does not reduce the cohesion metric, the algorithm terminates. In the eighth’s run, we see a similar phenomenon. Though the algorithm is able to find a refactoring opportunity, but since this refactoring has no impact on the cohesion metric, the algorithm terminates.

**Table 11:** Results of running KHC on our case study.

Experiment No.	Point	KHC Recommendation		
1	46	Keep it		
2	38	Keep it		
3	8	Keep it		
4	38	Keep it		
5	12	Keep it		
6	23	Move Message 6 to Class4 Move Message13 to Class1		
7	51	Keep it		
8	54	Move Message14 to Class3		
LCOM	0	0	0	1
Running Time	2 seconds			

In comparison with SA, HC is very inefficient in working in such difficult case study. SA’s strength in relaxation on the termination condition allowing for more iterations even if the refactoring opportunity is not found or the cohesion metric is not reduced, makes it more suitable than HC algorithm. Nevertheless, hybridizing HC with k-mean has its merits over running HC alone. As explained in the KSA section, hybridization directs HC to pick useful points (points with nonzero values as shown in Table 2).

### Experiment 2.B: KSA algorithm for Extract Message Refactoring

In this experiment, we show how KSA algorithm is applicable in refactoring extract message operation. Extract message operation is similar to the extract method in code refactoring. If the message has features that belong to more than one class, then we will create a set of new messages where each message contains features belonging to one class only. For instance, Message13 has two features belonging to Class1 and one feature belonging to Class2. In Move message operation, the algorithm will recommend moving this message from Class4 to Class1. In Extract Message operation, the algorithm will recommend creating a new message belonging to Class2 containing the features of Class2. We will name this message as Message13.1 to show that it is a new message extracted from the Message13. Table 12 shows the results:

**Table 12:** Results of running KSA for Extract Message Refactoring on our case study.

No.	Point	Representation	KSA Recommendation
1	24	Message 7, Class2	Keep Message 7 Create 7.1 to Class1
2	52	Message14, Class4	Move Message 14 to Class3 Create Message 14.1 to Class1
3	8	Message3, Class1	Keep it
4	8	Message3, Class1	Keep it
5	48	Message13, Class4	Move Message 13 to Class1 Create Message 13.1 to Class2
6	4	Message2, Class1	Keep it.
7	59	Message15, Class4	Keep it.
8	12	Message4, Class1	Keep it.
9	8	Message3, Class1	Keep it.
10	8	Message3, Class1	Keep it.
11	46	Message12, Class3	Keep it.
Parameter	Value	Recall	40%
Initial Temperature	100000000000.0	Precision	40%
Cooling Factor	temperature /10	Ratio	11/2 = 5.5
Threshold	Temperature > 1		
Running Time	4 Seconds		

The purpose of Experiment 3 is to answer RQ3: To what extent the search-based algorithm can be effective in a large case study with hundreds of refactoring opportunities?

### Experiment 3: Large case study

**Fig 10:**
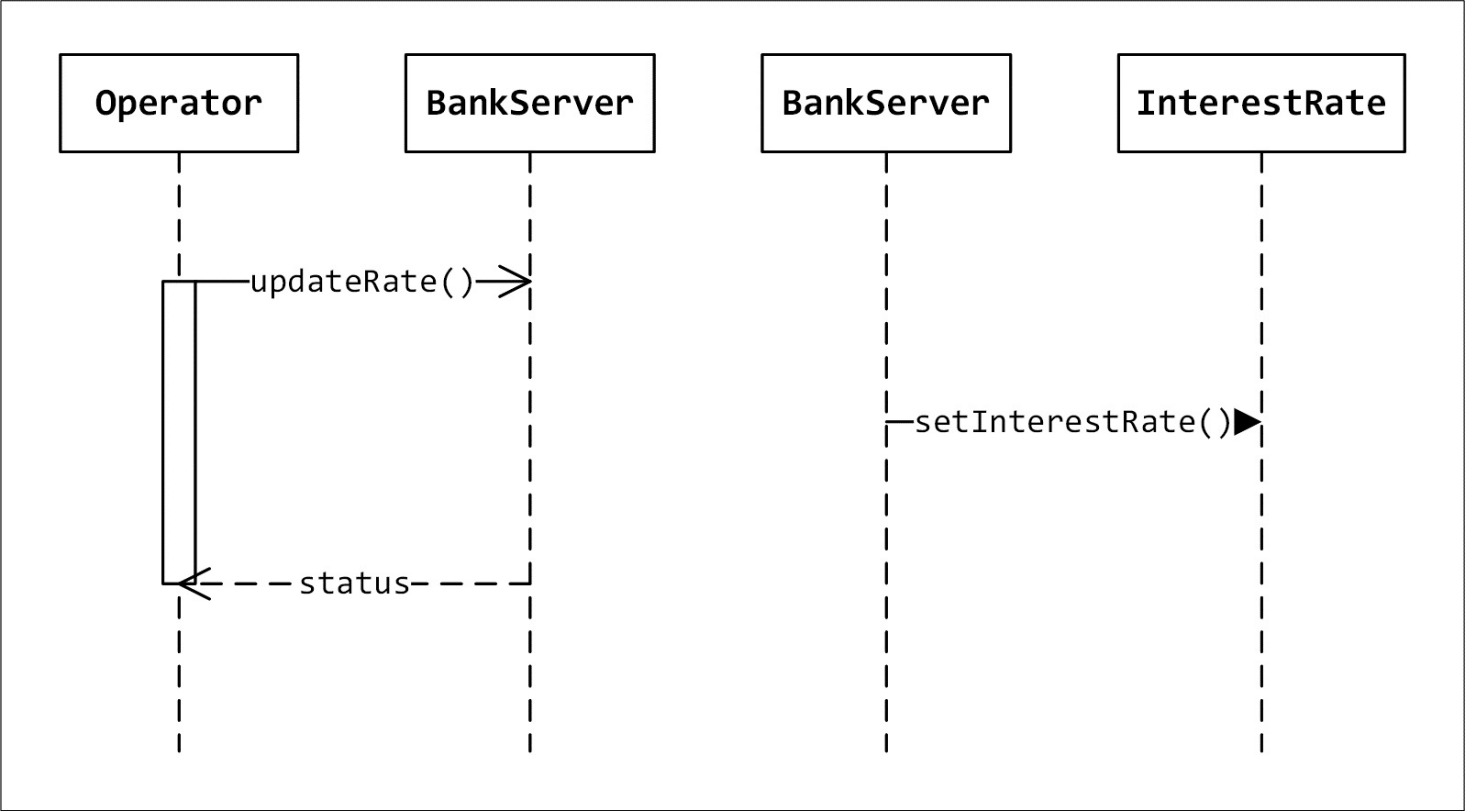
Original sequence diagram.

**Fig 11:**
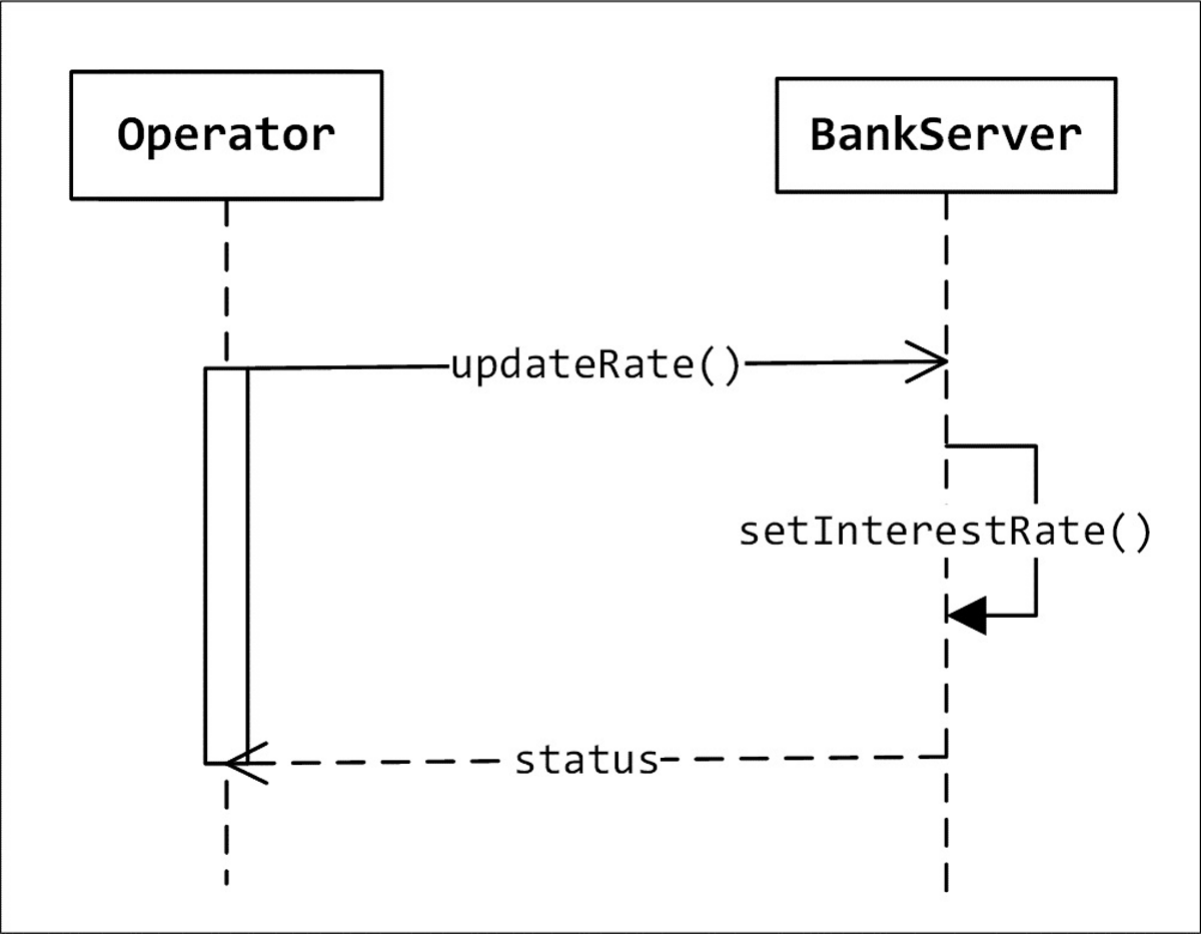
Refactored sequence diagram.

In this experiment, we used a large case study in order to see the effectiveness of a search-based algorithm namely: SA in refactoring sequence diagram. The anti-pattern of this case study is depicted in Fig 10 and the refactored diagram is depicted in Fig 11. Fig 10 shows that Bankserver class should communicate with InterestRate class to compute one function. It would be better if the function setInterestRate is inserted in the bankServer class and thus the class will reference its message. This operation will reduce the number of classes and will reduce the communication between different classes and increase the communication of loop messages which indicates high cohesion.

Using search-based for large case study, we applied SA to detect a sequence anti-pattern in a large case study. We ran it for 25 times and recorded the number of instances detected in each run. Then we calculated the average and the standard deviation of these runs as shown in Table 13.

**Table 13:** Number of anti-patterns detected in each run of SA.

No	Number of instances detected
1	636
2	645
3	648
4	585
5	664
6	707
7	648
8	701
9	575
10	634
11	680
12	612
13	656
14	686
15	664
16	703
17	696
18	679
19	633
20	647
21	667
22	636
23	663
24	655
25	702
Average	656.88
Standard Deviation	34.47018
Ratio of Average anti-pattern detection	656.88/1000 = 66%

The case study contains a ten thousand messages with a thousand anti-pattern instance. We set the number of non-optimizing iterations of SA to 997. So the number of iterations the SA goes in each run can be found by adding the number of detection to 997. For example, in the first run, SA goes for 636 + 997 = 1633 iteration.

In this experiment, we are not able to list all refactoring suggestions of the algorithm due to space limitations. For example, in the first run: there are 636 refactoring recommendations found by the algorithm. Since the objective of this experiment is to show the performance of SA in a large case study and since SA is working randomly, we calculated the average and the standard deviation of around 25 runs. Table 13 shows the details of this experiment. SA in average was able to find 66% of all instances in the case study.

### Threats to validity

There are a number of threats that affect the generalization of the results of this research. We ran our experiments on one case study. Other case studies with different characteristics may provide different results. However, we have adopted this case study from the literature in order to ensure the validity of our hybridized approach on a published case study. Another possible threat is that all algorithms are based on random initializations. This might lead to different results in each run. However, since our objective is to show the gain that can be acquired from the hybridization of data mining and search-based algorithms, we can assume that regardless of the initialized points, SA always will gain benefits from the results returned by the Kmeans algorithm.

The proposed hybridized algorithm implements a one-way communication from the Kmeans to the SA algorithm. This can aid the SA whenever it starts and can still have significance on the performance for small problems. However, for larger problems where SA runs for many iterations that modify the elements of the Kmeans clusters, there is no way for SA to seek help from the Kmeans algorithm to cluster the points again. For such problems, a full integration of the two algorithms with many levels of communication will be very helpful. This can be accomplished in future research.

In a larger problem, where there are many points that do not contribute to the results, clustering algorithms can be very helpful in passing only the good points to the SA algorithm. In our case, the maximum similarity is 2 and the minimum similarity is 1. The range is very short. However, in other problems, the range can span over a wide range between the maximum and the minimum similarity of features. The Kmeans algorithm can divide this range into different clusters, where SA can start from one cluster and move to a neighbor in another cluster. In addition, in a very large space, the resulting cluster from the Kmeans can be huge as well, with points that have different values resulting in a minor improvement to the SA algorithm.

Cohesion and coupling do not capture all aspects of software quality; however, since our objective is to show the effectiveness of the proposed algorithm using multiple competing metrics. Coupling and cohesion metrics are two competing metrics that have been used previously in other research and found to be effective in such context.

Search-based algorithms applied to software engineering introduce some construct validity as outlined by [46]. Construct validity is concerned whether the used measurements are relevant and meaningful to the study. One of these threats is the validity of the cost of executing the fitness function. Although, in our paper, we explained that KSA is able to reach to an optimal state of refactoring using 21 iterations, it is not clear how costly these iterations are with respect of time and resources.

Another possible threat is that we used software quality metrics along with precision and recall metrics to assess the effectiveness of the approach. We reason that KSA is better than SA due to the quality improvement measured by these metrics. However, these set of metrics have been previously used in many studies and have been shown to be relevant for such goal.

## Conclusion and future work

Data mining algorithms and search-based algorithms use different approaches to solve optimization problems. Clustering algorithms run based on the similarity between data, while search-based algorithms search the problem space using a guided fitness function. Both of these algorithms suffer from several limitations. In this paper, we have shown how two instances of these algorithms can communicate with each other to overcome some of the limitations and produce better results. The application of these algorithms has been tested on a sequence diagram refactoring case study. The experiments show the advantages that can be gained by hybridizing data mining and search-based algorithms on a software engineering domain problem.

Three runs were performed to show the results returned by the KSA algorithm. The KSA algorithm, after 21 iterations, was able to recommend all the necessary refactoring operations in order to reduce the LCOM2 value, thus increasing cohesion and reducing the coupling. In terms of recall and precision, if we allow the algorithm to run longer, going through more iterations, it is evident that it is going to retrieve or recall more recommended operations. However, it is interesting to note that the algorithm does not give any incorrect refactoring operation. In all three runs of the algorithm, precision is always equal to the recall value, implying that there is no false recommendation.

We compared our results in the case study with another publication. As they used a different coupling metric, a full comparison could not be made, yet their LCOM2 value decreased by 2 for one class while our algorithm decreases the LCOM2 value by 3.

Hybridizing SA with clustering shows interesting results. Hybridizing HC with clustering also provides good results. Running search based algorithm on another refactoring operation and on large case studies supports the merit of this approach.

Another future direction is to use other software metrics with advanced data mining algorithms and search-based algorithms.
